# Anonymous forensic evidence collection (AFC) after sexual offenses: a challenge in gynecological care—data from 13 years and 7 months at a University Hospital

**DOI:** 10.1007/s00404-026-08388-1

**Published:** 2026-03-17

**Authors:** Clarissa Herpel, Magdalena Bogus, Peter Mallmann, Sibylle Banaschak, Sebastian Ludwig

**Affiliations:** 1 Department of Obstetrics and Gynecology, University Hospital Cologne, University of Cologne, Faculty of Medicine, University Hospital Cologne, Kerpener Str. 34, 50931 Cologne, Germany; 2 Institute of Legal Medicine, University of Cologne, Faculty of Medicine, University Hospital Cologne, Melatengürtel 60-62, 50823 Cologne, Germany

**Keywords:** Sexual offense, Evidence collection, STI, Swabs

## Abstract

**Introduction:**

Anonymous (or confidential) forensic evidence collection (AFC; German: *Anonyme Spurensicherung* [ASS]) following sexual offenses plays a critical role in the initial care of affected individuals. Comprehensive execution of this procedure presents a significant challenge in the clinical routine care of gynecologists, as legal requirements, court-admissible documentation of injuries, and forensic evidence preservation often lead to uncertainty. The implementation of forensic evidence collection as a statutory health insurance benefit, following the Measles Protection Act (*Masernschutzgesetz*), has been effective in North Rhine-Westphalia since March 1, 2025, and is currently being formalized in contractual agreements. The objective of this study is to track the utilization of AFC, raise awareness of this topic, and identify common problems associated with its execution.

**Materials and methods:**

Systematic compilation of annual case numbers for anonymous forensic evidence collection (AFC) was performed from June 2011 to December 2024 at the Department of Obstetrics and Gynecology, University Hospital Cologne. This was followed by a statistical evaluation of the percentage increase and the annual mean values.

**Results:**

A total of 177 cases were documented at the University Hospital Cologne. Between 2012 and 2024, the annual case numbers increased from eight to 25 cases. The average number of cases examined annually during the observation period was 13.15. This corresponds to an average annual increase of approximately 17%, totaling 215% over 11 years, when considering 2012 as the first complete year for data analysis. Frequent issues encountered in clinical practice include improper storage (until transport to the Institute of Legal Medicine) and incorrect labeling of collected samples.

**Conclusions:**

The observed increase in the number of alleged victims of sexual violence requiring examination supports the health policy assumption that structured and correct forensic evidence collection is becoming increasingly vital. Adequate funding for the examination and the storage of evidence carriers is mandatory for long-term quality assurance. This is the only way to ensure optimal support and care for presumed victims in these stressful situations.

**Supplementary Information:**

The online version contains supplementary material available at 10.1007/s00404-026-08388-1.

## What does this study add to the clinical work

**Table:** 

The study adds several critical elements to clinical work by focusing on the practical challenges and policy changes surrounding Anonymous Forensic Evidence Collection (AFC). It highlights the clinical need and utilization with increasing case numbers, identifies specific error sources, discusses policy implementation and empathizes training mandates.	

## Introduction

Anonymous forensic evidence collection (AFC; also termed confidential forensic evidence collection, differing from region to region in Germany) following sexual violence plays a crucial role in the initial care of affected individuals. However, its execution represents a challenge for clinicians due to the legal implications and the psychological burden on the alleged victims. Consequently, a time-intensive and empathetic approach is mandatory.

Article 25 of the 2018 Istanbul Convention (the Council of Europe Convention on preventing and combating violence against women and domestic violence) stipulates that acute care (including confidential forensic evidence collection) for victims of sexual violence must be provided without repeated referrals and by adequately trained personnel. Both the (forensic) medical and the psychosocial care must be provided free of charge, be easily accessible, and be available in sufficient numbers [1, 2].

To ensure compliance with the requirements set out in the Istanbul Convention, a regulation for the financing of confidential/anonymous forensic evidence collection (§ 27 paragraph 1 sentence 6 SGB V) came into force in March 2020 as part of the Measles Protection Act (*Masernschutzgesetz*) [3]. This legislation designates confidential forensic evidence collection as a benefit covered by statutory health insurance and aims to identify deficits in current care structures and to standardize and improve provision accordingly [4].

## Current provision status

Anonymous or confidential forensic evidence collection offers victims the opportunity to undergo forensic collection of possible traces without time pressure or fear of legal consequences following sexual violence. Victims may subsequently decide whether or not to file a police report. While evidence collection, transport, and storage of samples are organized and funded by law enforcement agencies in the event of a report, care without a police report is currently organized and financed in Germany by specialized violence protection centers, projects, and organizations [2, 5].

There is a growing number of services that facilitate injury documentation, evidence collection, court-admissible documentation, and storage independent of reporting status. These services differ in various aspects, such as the duration of evidence storage [5, 6]. In some federal states, such as Mecklenburg-Vorpommern, forensic medicine centers are primarily responsible for confidential evidence collection (e.g., the victim protection clinic at the Institute of Legal Medicine in Rostock), whereas in North Rhine-Westphalia, for example, collaborations organized by separate organizations between gynecological clinics and institutes of legal medicine ensure provision (e.g., the “Anonymous Forensic Evidence Collection after Sexual Offenses (ASS)” model) [5, 7]. The digital documentation system iGOBSIS (Intelligent Violence Victim Evidence Collection and Information System) is expected to play a major role in the future execution of anonymous evidence collection in North Rhine-Westphalia [8].

## Quality standards

The German Society for Gynecology and Obstetrics (DGGG) has issued a statement outlining recommendations for the procedure of forensic evidence collection in the care of alleged victims of sexual violence [9]. These recommendations include specific guidance on the examination environment, the performing personnel, medical care, psychosocial management, and follow-up care. However, many of these requirements cannot currently be guaranteed nationwide due to current shortcomings in the care infrastructure in Germany [4, 10, 11]. Victims often experience long waiting times in clinics due to high workload in emergency services. Furthermore, they are often referred to other facilities (e.g., for HIV post-exposure prophylaxis) or for gynecological examination after an initial forensic medical examination [12–14]. The evidence collection, predominantly performed by gynecologists, often falls short of the required standards due to a lack of specialized knowledge and uncertainty regarding these aspects [12–19].

## Materials and methods

To track the utilization of confidential forensic evidence collection and identify common problems, systematic data collection of anonymous evidence collection has been conducted at the Department of Obstetrics and Gynecology, University Hospital Cologne, since mid-2011. Since no anonymous evidence collection was performed previously, no data collection occurred before this time.

Anonymous forensic evidence collection following sexual offenses has been performed at the University Hospital Cologne since June 2011 as part of “Anonymous Forensic Evidence Collection after Sexual Offenses (ASS)”—a project run by the “Emergency Call and Counseling for Raped Women” (*Notruf und Beratung für vergewaltigte Frauen*) for all participating Cologne hospitals. The data collection incorporates figures previously published in a 2019 study by the Institute of Legal Medicine at the University of Cologne [20]. This dataset has been supplemented with data up to and including 2024 and exclusively includes cases documented at the Department of Obstetrics and Gynecology, University Hospital Cologne. Evidence collection and initial care for female victims are carried out by medical staff of the Department of Obstetrics and Gynecology; male victims are cared for by the Evangelisches Klinikum Köln Weyertal (part of the University Hospital Cologne) and referred to that clinic if they present elsewhere. Within the ASS project, (consenting) adolescents aged 14 and over and adults are examined; children (< 14 years) are not included. The collected samples are forwarded to the Institute of Legal Medicine at the University Hospital Cologne, where they are stored for 10 years or until a potential police report is filed.

Data collected included the number of female victims who underwent anonymous evidence collection, their age, the number of subsequent police reports filed, and the average number of items of evidence per case. The proportion of collected samples that were blood and urine was also analyzed, along with the most frequently secured sample types.

It is important to note that this study only includes cases of anonymous forensic evidence collection. Cases of anonymous evidence collection at other Cologne hospitals participating in the “ASS” project were not considered. Cases of evidence collection with immediate police reporting are recorded by the State Criminal Investigation Offices (*Landeskriminalämter*) and forwarded to the Police Crime Statistics (PKS) at the Federal Criminal Police Office (BKA).

**Fig. 1:**
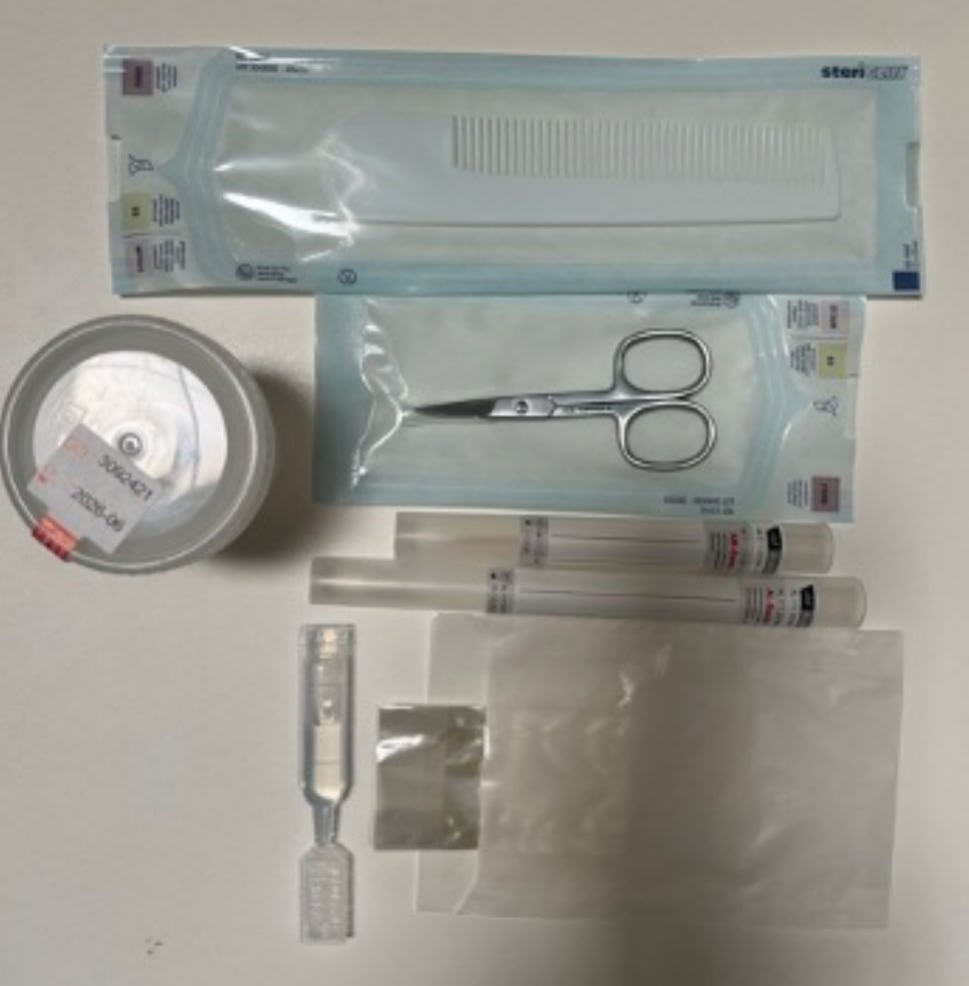
Examples of forensic evidence collection material. Shown are a urine container, nail scissors, a comb for combing pubic hair, a pointed swab for nail scrapings, a swab for forensic gynecological/other samples, bags for securing nail clippings, and saline solution for moistening the swab for the abrasion of dried traces.

In a case of anonymous evidence collection, the process first involves handwritten documentation of the patient history on the ASS documentation form (see Fig. 1 for examples of samples and the documentation form linked as “supplementary material”). This is followed by a physical and gynecological examination with history-dependent evidence collection, counseling, and documentation. Additionally, injuries are photographically documented, and worn clothing or objects (e.g., condoms) are secured in evidence bags. The written documentation form is kept in the patient’s medical record, and digital documentation is additionally entered into the hospital information system. The accompanying sheet for the evidence is anonymized using a cipher. The evidence is transported to the Institute of Legal Medicine on the following working day; the documentation form and images remain at the clinic. After 10 years, the evidence is destroyed, and the disposal date is documented via the sample registration system.

### Definition and recording of procedural errors

The data reported here is retrieved and documented annually as part of an annual statistic from the laboratory’s internal data and sample registration system. The evaluations were provided in tabular form to the Department of Obstetrics and Gynecology, University Hospital Cologne, by the Institute of Legal Medicine, University Hospital Cologne.

Procedural errors were defined as deviations from the ASS documentation and evidence handling protocol that required corrective action by the Institute of Legal Medicine or that could compromise trace analysis. These included incorrect labeling, improper packaging, incomplete sealing, inappropriate sample type usage, and submission of non-anonymized documentation forms.

Errors were recorded within the Institute of Legal Medicine’s internal registration system when identified during evidence intake. However, a prospective standardized audit protocol with predefined quantitative endpoints was not implemented during the study period.

### Catchment context

The University Hospital Cologne is a tertiary referral center serving the metropolitan area of Cologne and surrounding regions. As mentioned above, AFC is provided within the framework of the ASS network, which includes several participating hospitals in Cologne. The present analysis includes only cases managed at the Department of Obstetrics and Gynecology at the University Hospital Cologne. Population-based denominators (e.g., total female population in the catchment area or total number of sexual offenses in the region) were not available for linkage; therefore, incidence or prevalence rates cannot be calculated.

### Data completeness

Annual AFC case numbers were extracted from the Institute of Legal Medicine’s sample registration system. No missing data occurred for the primary outcome (annual case count). Information on subsequent police reporting was available only if voluntarily communicated to the clinic; therefore, reporting rates may be underestimated.

## Results

**Fig. 2:**
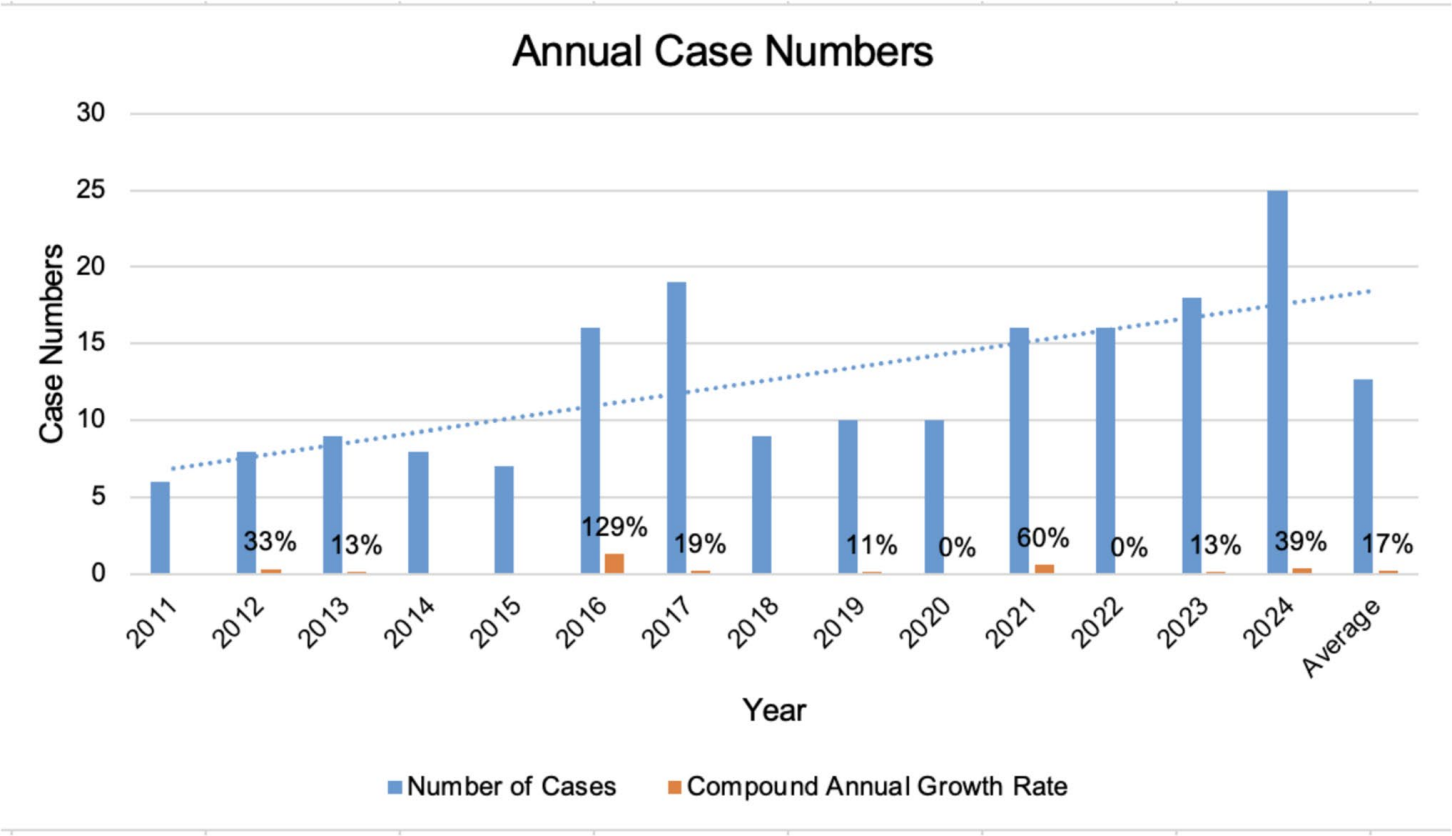
This figure shows the annual number of victims who utilized AFC at the Department of Obstetrics and Gynecology in Cologne. An upward trend has been recorded since the project’s inception in 2011. The orange bars show the percentage of the annual increase.

A total of 177 cases of anonymous forensic evidence collection were recorded at the Department of Obstetrics and Gynecology, University Hospital Cologne, between June 2011 and the end of 2024. The number of annual cases increased by 317% during this period. However, there were some years with a decrease in case numbers (e.g., a drop from 19 to nine annual cases between 2017 and 2018) (Table 1, Fig. 2).

**Table 1:** This table displays the annual number of cases of AFC, as well as the compound annual growth rate in cases numbers

Year	Number of cases	Compound annual growth rate (CAGR)
2011	6	
2012	8	33%
2013	9	13%
2014	8	− 11%
2015	7	− 13%
2016	16	129%
2017	19	19%
2018	9	− 53%
2019	10	11%
2020	10	0%
2021	16	60%
2022	16	0%
2023	18	13%
2024	25	39%

To quantify the temporal trend in annual case numbers, the compound annual growth rate (CAGR) was calculated using the first complete observation year (2012) and the most recent year (2024). The CAGR was computed according to the formula:

$$CAGR = \left( {\frac{{N_{2024} }}{{N_{2012} }}} \right)^{1/12} - 1$$

where ${N}_{2024}$ and ${N}_{2012}$ represent the annual case numbers in the respective years.

Because evidence collection began in June 2011, 2012 was considered the first complete year for trend analysis. The calculated CAGR represents an average growth rate over time and does not imply linear yearly increases.

Between 2012 (first complete year; 8 cases) and 2024 (25 cases), the annual case number increased by 213% in absolute terms. The calculated compound annual growth rate over this period was approximately 17%. Given the small annual case numbers, this figure should be interpreted as a descriptive summary measure rather than as evidence of a statistically confirmed linear trend.

The annual case numbers show that the average monthly case number fluctuates between 0 and approximately two victims. The highest number of cases was recorded in 2024 (25 cases), and the lowest in 2011 with six cases, followed by 2015 with seven cases. The strongest percentage increase in cases was from 2015 to 2016, at 129% (from 7 to 16 annual cases). An average of 12.64 cases is recorded annually.

**Fig. 3:**
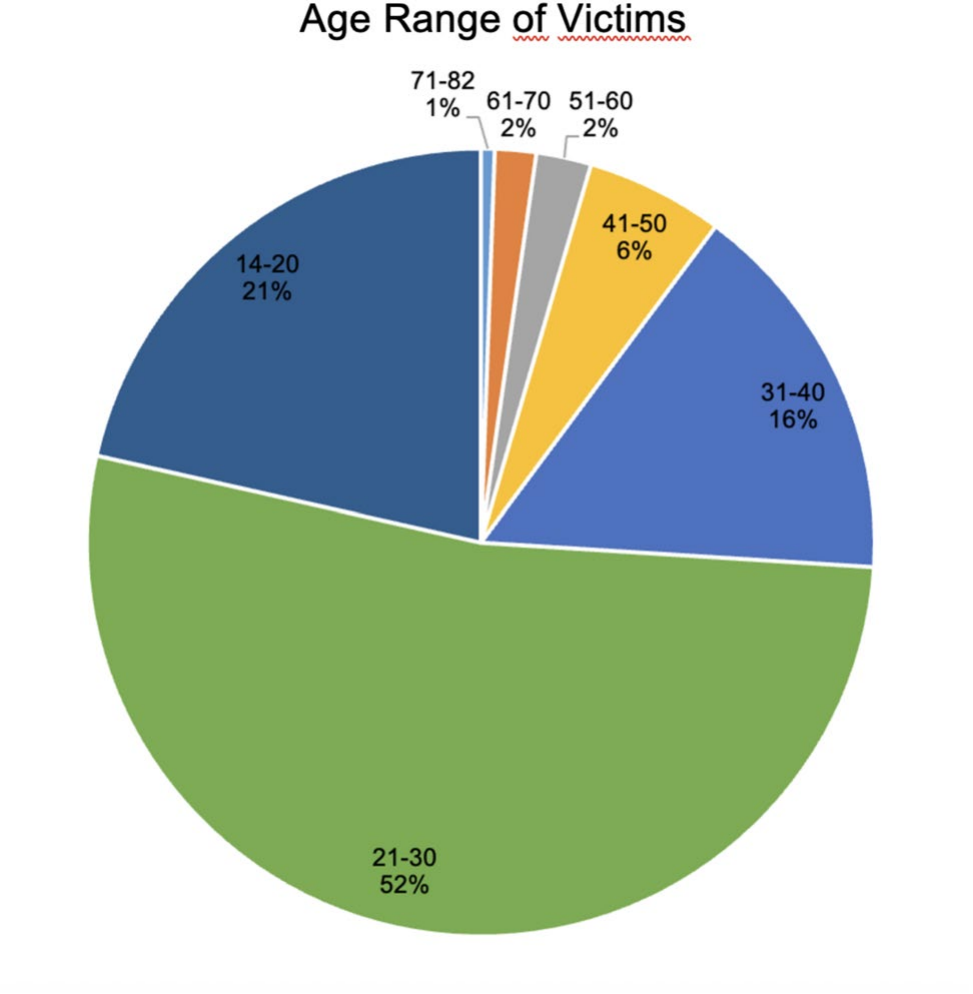
This figure shows the age distribution of the affected women who utilized AFC at the Department of Obstetrics and Gynecology in Cologne since 2011. Age is presented in years, and the proportion is indicated as a percentage of the total case number.

The youngest victim was 14 years old, and the oldest was 82 years old (Table 2). The majority (53%) of victims were between 21 and 30 years old at the time of the incident, followed by the age range between 14 and 20 years (Fig. 3), with 15 of the examined victims being between 14 and 18 years old. Only women were examined at the Department of Obstetrics and Gynecology.

**Table 2:** This table displays the number of cases of AFC per age range, as well as the percentage of this age range per total number of cases

Age range (Years)	Number of cases	Percent of all registered cases
71–82	1	1%
61–70	3	2%
51–60	4	2%
41–50	10	6%
31–40	28	16%
21–30	93	53%
14–20	38	21%
Min Age	14	
Max Age	82

An average of 6.61 samples or items are collected per case. In approximately 1% of cases, urine and/or blood samples were also taken. Since 2011, 15 cases resulted in a subsequent police report, which is 8.5% of the cases. Reports were filed from one day up to seven months after the evidence collection. The most frequently collected items are forensic gynecological swabs (swabs from the vulva/introitus, anterior and posterior fornix, and the cervix), followed by nail clippings or scrapings. The third most frequent items secured were clothing, followed by buccal swabs (either for comparison DNA or in cases indicating oral intercourse) and combed pubic hair.

Approximately 10% of submitted kits required return shipment due to inclusion of identifiable documentation materials or incomplete anonymization. Other procedural deviations (e.g., labeling errors or improper packaging) were recorded qualitatively during internal annual reviews but were not systematically quantified throughout the entire observation period.

### Limitations of evidence collection—the balance between quality collection, documentation, and adequate trace analysis

The Institute of Legal Medicine at the University Hospital Cologne records conspicuous or flawed samples and incorrectly submitted documentation forms for each case, which are also evaluated annually. The staff handling the evidence at the Institute of Legal Medicine provide feedback to the gynecological team. These issues are discussed during regular training sessions within the gynecological team. The sources of error include the following points, which either lead to increased time expenditure, violate data protection, or render the collected samples unusable for forensic analysis and potential prosecution:

Jeopardizing Potential Trace Analysis by Having to Destroy Secured Samples or Evidence:

- Poorly legible, carelessly applied labeling on swab tubes.
- Labeled but detached adhesive labels—the swab can no longer be matched to its source.
- Unrefrigerated blood and unfrozen urine samples.
- Fingernails are not individually packaged in designated glassine envelopes but are placed together in a single bag.
- Unsealed and non-tamper-evident collection bags, which may be subject to manipulation during transport.

Work efficiency and data protection:

- Shipping the examination form and/or recorded image material to the storage institute. This absolutely necessitates a return shipment and occurs in over 10% of submissions overall.

Diligence:

- Incorrect swab types used (e.g., nail scraping swabs for gynecological samples).

## Discussion

Our analysis records an overall increase in the case numbers of anonymous forensic evidence collection at the University Hospital Cologne since 2011. It must be considered that the data is based on a monocentric collection with a low overall case number (averaging 0 to 2 cases per month). The representativeness of this data and, consequently, its generalizability to the overall situation in Germany is questionable, and only assumptions can be made.

The presented data represent service utilization within a single tertiary care center and must not be interpreted as incidence data for sexual violence in Cologne or Germany. Without defined denominators or linkage to police or population data, only descriptive trends in service use can be reported.

Ultimately, it remains unclear whether the observed increase in case numbers is due to a surge in cases of sexual violence or to increasing public awareness of the availability of evidence collection services. Consequently, the service is either being utilized more frequently by the population or is more readily available as an option for victims when needed.

Various campaigns, such as those by the association “Frauen gegen Gewalt e. V.” in December 2023 in Cologne subway stations, are increasing public knowledge about confidential evidence collection. Furthermore, an increase in sexual offenses recorded by the Cologne Police Headquarters has been noted [20–23]. This suggests an increased willingness to report within the population.

Temporal fluctuations in annual case numbers coincided with societal and legislative events, including the reform of §177 StGB (2016), the Cologne New Year’s Eve incidents, and the COVID-19 pandemic [20]. However, no formal time-series or interrupted trend analysis was performed. Therefore, causal relationships between these events and AFC utilization cannot be established.

These contextual considerations are presented as possible explanatory hypotheses rather than as analytically verified associations.

### Demographic data

The other data suggest that evidence collection following sexual offenses is most frequently accessed by younger women, but ultimately, all age groups can be affected. Whether younger women are more likely to seek evidence collection than, for example, older women, or whether the dark figure of sexual offenses is higher in certain age groups remains unclear [21–24]. It is also plausible that the observed increased utilization of confidential evidence collection among younger women is due to a potentially greater range of movement, more frequent changes in partners, a more open approach to the topic, and awareness of information sources regarding AFC.

The utilization of the option for anonymous or confidential evidence collection is increasing, and it is evident that a critical examination of the resulting demands on medical personnel is not solely at the level of gynecology but also from a forensic technical perspective.

The problems reported back to the Department of Obstetrics and Gynecology concerning evidence collection may suggest insufficient training of staff. Specific attention must be paid to the highly sensitive handling of case documents. The violation of the anonymity principle through improper handling of the documentation forms is unacceptable. Only when the medical staff is released from the obligation of confidentiality upon the filing of a police report is the transmission of personal data to the investigating authorities and potentially to the personnel at the storage and examination facility permitted. Furthermore, the necessary process of reversing the documentation between the Institute of Legal Medicine and the Department of Obstetrics and Gynecology results in considerable extra time expenditure. Attempts have been made to minimize this problem. Nevertheless, the documentation forms containing the patients’ personal data are included in approximately 10% of the submitted evidence collection kits. Problems during sample transport or storage can be easily resolved through regular staff training. The use of the wrong sample type or careless labeling may also be due to a lack of time resources in emergency service provision. Regular training sessions by the association “Emergency Call and Counseling for Raped Women—Women Against Violence e. V.,” the Institute of Legal Medicine at the University Hospital Cologne, and experienced medical staff take place 1–2 times a year at the Department of Obstetrics and Gynecology. However, the turnover of performing personnel, with varying degrees of experience in handling evidence collection, can also lead to errors in evidence preservation. Therefore, regular training and participation in annual meetings are essential. Anonymous evidence collection is carried out according to the instructions on the documentation form, based on the training provided by the Institute of Legal Medicine and the association “Emergency Call and Counseling for Raped Women—Women Against Violence e. V.”

However, sensitive handling of trace material and proper evidence preservation are increasingly being incorporated into the training of medical personnel. Information on new, highly sensitive analysis methods for processing traces and potentially determining the trace source is conveyed here as part of teaching, creating the necessary change in perspective for securing and handling trace material.

### Significance for healthcare provision

With rising case numbers of sexual offenses, the care of alleged victims is gaining increasing importance in clinical practice. If the current trend continues, evidence collection will increasingly take center stage in clinical routines. Physicians are often the first point of contact for alleged victims. Uniform, comprehensive provision and financial security that meet the requirements of the DGGG, the World Health Organization (WHO), and the Istanbul Convention are urgently required. Consequently, regular training of the personnel performing the procedures is also important. This can convey aspects of court-admissible injury documentation and evidence collection, and minimize uncertainties and potential sources of error. In future, digital documentation will play a greater role. In this context, workflow steps in form-based processes can be recorded in a valid and thus measurable manner.

### Limitations

The conducted study has several limitations. Above all, the monocentric nature of the data collection and the consequently low case numbers significantly limit the data’s representativeness. The limited collection of sociodemographic data also significantly restricts the validity of the findings. Error frequencies were not prospectively collected using a standardized quality monitoring protocol; therefore, the magnitude of specific procedural deviations cannot be precisely quantified. The small number of variables collected represents a further limitation of this work.

This monocentric descriptive analysis demonstrates increasing utilization of confidential forensic evidence collection at a tertiary gynecological center over a 13-year period. The findings highlight structural and procedural challenges in clinical implementation. However, due to the absence of population denominators and analytic modeling, conclusions regarding epidemiological trends or policy effects cannot be drawn. Future multicenter studies with standardized quality indicators and defined catchment denominators are required to better characterize health service provision in this field.

### Future research approaches

In future research projects, the collection of additional data points, such as the time/point of evidence collection (during emergency duty vs. regular daytime operations), the average duration of care per case, or the costs incurred by the clinic for evidence collection, would provide a more concrete picture of the care situation. A complete compilation of all confidential evidence collection services in Germany and their financing would also contribute to providing an overview. This could potentially be achieved through an association of gynecological clinics and/or practices [23, 25]. The establishment of an inter-clinic/practice working group would contribute to health services research for victims of sexual violence, as a great deal of work is currently being done by women’s associations and the institutes of legal medicine, particularly regarding the implementation of the Measles Protection Act.

## Conclusion

The observed increase in AFC utilization over time may reflect improved awareness, accessibility, or structural implementation of confidential forensic services. However, causal inferences regarding changes in the prevalence of sexual violence cannot be drawn from these data. Frequent errors during the execution of evidence collection include incorrect labeling or faulty transport of the evidence. Regular training can contribute to improving these aspects.

In this context, the 2020 amendment to the Measles Protection Act represents a significant step forward. However, the regional heterogeneity of care services, insufficient staff training, a lack of capacity in clinical routines, and incomplete financing remain major obstacles to adequate care for alleged victims. The development of a new guideline by the German Society for Gynecology and Obstetrics, as well as, for example, the introduction of iGOBSIS, are important steps toward improving provision. Ultimately, however, the goal must be to prevent sexual violence from occurring in the first place, which requires more prevention work in society.

## Supplementary Information

Below is the link to the electronic supplementary material.

Supplementary file1 Link to the documentation and instruction form for evidence collection for the “ASS” project of the association Notruf und Beratung für vergewaltigte Frauen—Frauen gegen Gewalt e.V. (Emergency Call and Counseling for Raped Women – Women Against Violence registered association). (PDF 450 KB)

## Data Availability

No datasets were generated or analyzed during the current study.
