# Supplementary material for: Anonymous forensic evidence collection (AFC) after sexual offenses: a challenge in gynecological care—data from 13 years and 7 months at a University Hospital
Source: Arch Gynecol Obstet. 2026 Mar 17;313(1):131. doi: 10.1007/s00404-026-08388-1 (PMC12996375; doi:10.1007/s00404-026-08388-1)
Supplement: Supplementary file 1 — Supplementary file1 Link to the documentation and instruction form for evidence collection for the “ASS” project of the association Notruf und Beratung für vergewaltigte Frauen—Frauen gegen Gewalt e.V. (Emergency Call and Counseling for Raped Women – Women Against Violence registered association). (PDF 450 KB) [file 404_2026_8388_MOESM1_ESM.pdf]

# Dokumentationsbogen

# ASS – Anonyme Spurensicherung nach Sexualstraftaten

(überarbeitete Version Dezember 2022)

**Im Dokubogen enthalten:**

- 1 Merkblatt Spurensicherung (bitte vor Untersuchung durchlesen!)
- 1 ärztlicher Untersuchungsbericht mit Ganzkörperschema – **zum Verbleib in Klinik !**
- 2 Erklärungen über die vorläufige Nichtanzeige der Straftat
- 1 Begleitschreiben für Asservate **– zum Versand in Rechtsmedizin**

**dazu:**

- 1 Spurensicherungsset Sexualstraftaten NRW
  → bitte separate Inhaltsbeschreibung beachten
- 1 kleine und 1 große Packpapiertüte
- 1 Siegel-Aufkleber
- 1 Info-Flyer zur Weitergabe an die betroffene Person

## Wichtig – bitte vorab lesen:

*Seit April 2021 werden die neuen, zentralen NRW-Sets zusammen mit dem vorliegenden Dokumentationsbogen und dem zusätzlichen Material (s.o.) benutzt.*

***1 Plastiktüte = 1 Gesamtset!***

*Die NRW-Sets sind sehr umfangreich, u.a. sind auch Blutentnahme-Sets und Urinbecher enthalten.*

Gesichertes **Spurenmaterial**, inkl. ggf. entnommener Blut- und/oder Urinproben, mit ausgefülltem Begleitschreiben schnellstmöglich an:

**Institut für Rechtsmedizin der Uniklinik Köln**
**Melatengürtel 60-62**
**50823 Köln**
(Annahmezeiten: Mo-Fr 8-16 Uhr)

*Versand von **Blut- und Urinproben** an die Rechtsmedizin*
*→ gefrorene/gekühlte Lagerung und Transport!*
*→ **Vermerk** auf Umschlag und Info an Transport!*

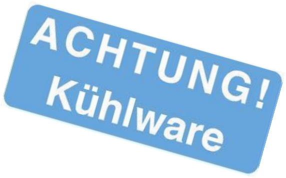


**Dokubogen + Fotos NICHT mit in die Rechtsmedizin senden !**

→ verbleibt bis auf Abruf in der Krankenakte des/der Betroffenen

Vor Untersuchung durchlesen !

# *Merkblatt zu ASS*
# *Anonyme Spurensicherung nach Sexualstraftat*

Im Folgenden erhalten Sie einige Hinweise, wie Sie als Arzt/ Ärztin eine solche Spurensicherung gewährleisten können und welche Schritte dazu erforderlich sind. Sollten Sie Fragen zum Verfahren oder grundsätzlich zum Thema sexualisierte Gewalt haben, können Sie sich an das Institut für Rechtsmedizin der Uniklinik Köln oder die ASS-Koordinatorin beim Notruf für vergewaltigte Frauen e.V. (siehe Deckblatt) wenden.

## 1. Chiffre

Jede zu untersuchende Person erhält eine Chiffre, die gewährleistet, dass das Opfer anonym bleibt und die gesicherten Spuren trotzdem eindeutig zugeordnet werden können. Die Chiffre setzt sich aus dem Anfangsbuchstaben des Nachnamens, dem 6-stelligen Geburtsdatum und dem 6-stelligen Untersuchungsdatum zusammen.

Beispiel: Eva Mustermann, geb. 22.02.1965; Untersuchung war am 16.08.2005
Chiffre: M 220265 / 160805

**Die Chiffre ist in die vorgegebenen Felder und auf die Aufkleber einzutragen.**

## 2. Grundlagen

Die Spurensicherung, die Sie im Rahmen Ihrer Untersuchung vornehmen, dient in erster Linie der Sicherung von DNA-Spuren.
Der **Umfang der Spurensicherung** bei der betroffenen Person richtet sich nach den Angaben, die zum Täterkontakt gemacht werden. Über Anzahl und Ort der Abstriche entscheiden Sie je nach Vorgeschichte.

So ist in einem Vorgespräch zu klären:

- wurde penetriert (vaginal, anal, oral)?
- wurde ejakuliert (vaginal, anal, oral)?
- wurde Speichel übertragen (lecken, küssen o. ä.)?
- hat sich die betroffene Person durch Kratzen gewehrt?
- welche Bekleidung wurde von der betroffenen Person zur Tatzeit getragen?
- gibt es den Verdacht auf den Einsatz von best. Substanzen (z.B. K.o.-Tropfen)?

**BITTE niemals versuchen, das Opfer zu einer Anzeige bei der Polizei zu überreden! Man kann ganz neutral darauf hinweisen, dass es diese Möglichkeit ebenfalls gibt, sollte dann aber die Entscheidung zur ASS respektieren und keinen Druck ausüben (auch wenn man privat eine andere Haltung vertritt).**

## 3. Spurensicherung

<u>Wichtig:</u> **Jedes Spurenmaterial und jeden Abstrichtupfer gut lesbar beschriften, ggf. mit Entnahme-Ort !**

- Saubere Handschuhe und ggf. Mundschutz bei der Spurensicherung tragen. Sprechen, Husten, Niesen in Spurennähe unbedingt unterlassen.

- **Zur Tatzeit getragene Kleidung**, auf der Sekretantragungen des Täters zu erwarten sind (z. B. Slip), **im Original sichern** und separat in kleine **Papiertüte** verpacken.

- **Sekretspuren** (Blut, Speichel, Sperma, Vaginalsekret) **mit Abstrichtupfern** sichern.

- **Eingetrocknete Sekretspuren** mit angefeuchteten (0,9% sterile Kochsalzlösung) Abstrichtupfern sichern.

  Sachverhaltsabhängig sind Abstriche <u>zu sichern:</u>

  - **vaginal** 3-fach<br>(mindestens vorderes und hinteres Scheidengewölbe, ggf. Cervix)
  - **oral, anal** jeweils 1x
    - Vermerk bei oralen Abstrichen, ob es sich um eine Vergleichsprobe der/des Betroffenen oder ein Asservat wg. Oralverkehr handelt

  <u>oder/und</u>**:**

  **Körperregionen abzureiben** (hierbei müssen die Abstrichtupfer mit 0.9 % steriler Kochsalzlösung angefeuchtet werden).

*Die Abstrichtupfer sind selbsttrocknend und können sofort verschlossen werden.*

- **Schambehaarung** ist mit zuvor desinfiziertem Kamm auszukämmen. Ausgekämmte Haare und Kamm werden in einen Umschlag verbracht.

- **Fingernagelschnitte** nach Händen getrennt in je einen Umschlag einbringen und diesen verschließen. Wenn es aufgrund des Tatherganges nötig erscheint, Abriebe unter jedem Fingernagel einzeln sichern (in den neuen Sets sind hierfür separate spezielle Abstrichtupfer vorhanden).

- **Fotografische Dokumentation** etwaiger Verletzungen mit Winkellineal (Achtung: Bildmaterial nicht in die Rechtsmedizin schicken, sondern in der Krankenakte belassen)

## 4. Versand in die Rechtsmedizin (siehe Deckblatt)

Krankenakte

## Ärztlicher Untersuchungsbericht zur körperlichen Untersuchung

## 1. Allgemeine Angaben

**1.1 Personaldaten des Opfers**

Familien-/ Geburtsname:

Vorname:

Geschlecht weiblich / männlich / divers

Geburtsdatum/ -ort:

Gesetzl. Vertreter*in (falls zutreffend):

Chiffre: .......... .............................. / ..............................

**1.2 Behandelnde Ärzt*innen (falls bekannt)**

Hausarzt/ -ärztin:

Gynäkologe/-in:

**1.3 Untersuchungsdaten**

Name des Arztes / der Ärztin (bitte Blockbuchstaben):

Name des Krankenhauses:

Ort / Datum / Uhrzeit:

## 2. Sachverhalt

Stichwortartige Schilderung des Sachverhalts (wenn nötig, Rückseite benutzen):

Wer

Was

Wann

Wo

Wie

Was wurde nach der Tat getan?
(geduscht, gewaschen, Kleidung gewechselt?)

Bei Angriff gegen den Hals (Drosseln/ Würgen):
Bewusstlosigkeit? Urin/Kotabgang? Petechien (Augenlider, -bindehäute, Mundschleimhaut, Haut hinter den Ohren), Schluckbeschwerden/ Heiserkeit?

## 3. Allgemeine Anamnese

Vorbestehende Krankheiten/ Leiden?

Behinderungen?

Medikamentöse Therapie?

Verletzungen?

## 4. Tatbezogene Anamnese

| **4.1 Deliktbezogene Schmerzen / sonstige Angaben** |
|---|
| |

| **4.2** | **Nur bei Vergewaltigung auszufüllen** |
|---|---|
| | **Verhütungsmittel (nach Angaben des Opfers)**<br>Benutzten **Täter oder Opfer** zur Tatzeit Verhütungsmittel?<br>ja welche?<br>nein |
| | **Schwangerschaft**<br>ja welche Woche?<br>nein |
| | **Innerhalb der letzten 7 Tage einverständlicher Geschlechtsverkehr?**<br>ja a) wann?<br>b) mit wem?<br>c) Verhütungsmittel<br>ja nein<br>nein<br>keine Angaben |
| | **letzte Menstruation**<br>wann? |

# 5. Untersuchungsbefund (bitte ggf. Rückseite benutzen)

**5.1 Physischer und psychischer Gesamteindruck**
**(Vorsicht! Objektiv sein, keine Wertungen vornehmen)**
ergänzende Angaben in Stichworten

| | |
|---|---|
| Bewusstseinslage: | klar, benommen, somnolent |
| Denkablauf: | geordnet, sprunghaft, perseverierend, fixiert, phobisch, zwanghaft paranoid, überwertig, ideenflüchtig, weitschweifig, zerfahren |
| Stimmung: | ausgeglichen, heiter, gereizt, ängstlich, aggressiv, traurig |
| Orientiertheit: | zeitlich: gestört, nicht gestört / örtlich: gestört, nicht gestört |
| Kontakt: | unauffällig, distanzlos, überangepasst, scheu, ablehnend |
| Gedächtnis: | unauffällig, amnestisch, lückenhaft, punktuell, zerstreut, hypermnestisch |

**5.1.1 Anhaltspunkte für Alkohol-, Medikamenten- oder Drogeneinfluss (evtl. auch sog. „K.o.-Tropfen“)**

- eigene Einschätzung und

- Verletzte auch dazu befragen

**5.1.2 Blutentnahme / Urinprobe erforderlich?**

Durchführung bitte in 6.3 / 6.4 dokumentieren!

| 5.2 | **Gynäkologischer Befund** |
|---|---|
| | Spekulum-Untersuchung: |

| | |
|---|---|
| | Befund des Hymenringes: |
| | Positive Hinweise für Analverkehr (hinterlässt nicht immer Verletzungszeichen): |
| | Gynäkologischer Tastbefund: |
| | Sichtbare Verletzungen des Genital-/ Analbereiches:<br>(Festgestellte Verletzungen bitte beschreiben und evtl. vorhandene Hämatome in ihrer Farbtönung angeben, Verschmutzungen beschreiben)<br><br>a) Genitalbereich:<br><br>b) Analbereich: |
| | Epikrise: |
| **5.3** | **Allgemeine körperliche Untersuchung**<br>Verletzungen auch registrieren, wenn nicht behandlungsbedürftig. Erst beschreiben – dann interpretieren.<br>Genaue Lokalisation, Art, Ausdehnung, Alter, mögliche Ursachen? Hautabschürfungen, -durchtrennungen, -unterblutungen? Schwellungen, verdächtige Fesselungs-, Festhaltespuren (Handgelenke, Fußknöchel)? Abwehrspuren? Widerlagerverletzungen? Bissspuren? Frische oder ältere Injektionsstellen? Auffällige Narben? etc.<br>**z.B. Hämatome: genaue Lokalisation – rechte Oberarminnenseite, körpernahes Drittel; Farbe – blaues Hämatom; Form – rundlich; Größe – 1 cm Durchmesser** |

| |
|---|
| **Kopf** |
| **Hals,** insbesondere durch Drosseln/ Würgen |
| **Brust** |
| **Bauch** |
| **Rücken** |
| **Arme** |

**6.3 Blutentnahme**
**zur Blutalkoholbestimmung, zum Schwangerschaftsnachweis oder bei Verdacht auf K.o.-Tropfen**

**ja** **Datum / Uhrzeit :**

**durch :**

**nein**

**6.4 ggf. Urinprobe**
***(bei Verdacht auf Einnahme von Betäubungsmitteln / Medikamenten oder Verabreichung von K.o.-Tropfen)***

**ja** **nein**

**6.5 Sicherung von Mundsekret zum Zwecke des DNA Vergleiches**
***Abrieb des inneren Wangenbereiches mittels Stieltupfer.***

**6.6. Auffälligkeiten an der Bekleidung, insbesondere Unterbekleidung**
***Im Hinblick auf Spurensicherung, z.B. Beschädigungen, Schmutz, Blut, Sekrete***

## 7. Bemerkungen: Untersuchung/ Behandlung

**Insbesondere Untersuchung auf: HIV, Hepatitis C und B, Gonorrhöe, Chlamydien, Lues etc.**

***Auch: verabreichte Medikamente, Empfehlung weiterer fachärztlicher Untersuchung***

**Über die Spurensicherung hinausgehende Untersuchungen müssen mit der Krankenkasse oder der/dem Patient*in selbst abgerechnet werden.**

**Ende der Untersuchung: Uhr**

.......................................................... ..........................................................

Ort, Datum Unterschrift

Arztstempel

# - Original für Krankenhaus –

## ***Erklärung***

**Chiffre: ........ .................... / ....................**
(bestehend aus Anfangsbuchstaben Nachname, 6-stelligem Geburtsdatum und 6-stelligem Untersuchungsdatum)

Ich habe heute, am (Untersuchungsdatum) ..........................................,

das Krankenhaus (ggf. Stempel) ………………………...............................................

.......................................................................................................................

aufgesucht, um mich nach einer Sexualstraftat untersuchen zu lassen.

Zurzeit möchte ich noch keine Anzeige erstatten.

Ich wünsche, dass das entnommene Spurenmaterial der Rechtsmedizin Köln in anonymisierter Form übersandt wird, d.h. ohne dass mein Name dort bekannt wird. Das Spurenmaterial wird dort für die Dauer von 10 Jahren aufbewahrt. Sollte ich innerhalb dieser Zeit keine Anzeige erstatten, wird das Spurenmaterial vernichtet. Sollte ich mich innerhalb der Zeit zu einer Anzeigenerstattung entschließen, wird das Spurenmaterial zu den Akten genommen.

Ich bin ausdrücklich darauf hingewiesen worden, dass eine Beweisführung nach Vernichtung des Spurenmaterials erheblich erschwert sein wird.

.......................................................... ..........................................................
Ort, Datum Unterschrift

Arztstempel

# - Zum Verbleib bei dem/der Patient*in -

## ***Erklärung***

**Chiffre: ........ .................... / ....................**
(bestehend aus Anfangsbuchstaben Nachname, 6-stelligem Geburtsdatum und 6-stelligem Untersuchungsdatum)

Ich habe heute, am (Untersuchungsdatum) .........................................,

das Krankenhaus (ggf. Stempel) ………………………..............................................

.........................................................................................................................

aufgesucht, um mich nach einer Sexualstraftat untersuchen zu lassen.

Zurzeit möchte ich noch keine Anzeige erstatten.

Ich wünsche, dass das entnommene Spurenmaterial der Rechtsmedizin Köln in anonymisierter Form übersandt wird, d.h. ohne dass mein Name dort bekannt wird. Das Spurenmaterial wird dort für die Dauer von 10 Jahren aufbewahrt. Sollte ich innerhalb dieser Zeit keine Anzeige erstatten, wird das Spurenmaterial vernichtet. Sollte ich mich innerhalb der Zeit zu einer Anzeigenerstattung entschließen, wird das Spurenmaterial zu den Akten genommen.

Ich bin ausdrücklich darauf hingewiesen worden, dass eine Beweisführung nach Vernichtung des Spurenmaterials erheblich erschwert sein wird.

......................................................... .........................................................
Ort, Datum Unterschrift

Arztstempel
